# Zearalenone Biotransformation by Tibetan Plateau-Derived Yeast *Hannaella zeae*: Biological Pattern Elucidation, Metabolite Safety, and Environmental Tolerance

**DOI:** 10.3390/toxins18010002

**Published:** 2025-12-19

**Authors:** Chenxiaoye Yang, Jiali Hu, Disha Jiang, Geng Ni, Changling Wu, Qiang Chu, Sergei A. Eremin, Liliya I. Mukhametova, Xiaofang Guo, Ji De, Xingquan Liu, Hao Hu

**Affiliations:** 1 College of Food and Health, Zhejiang Agriculture and Forestry University, Hangzhou 311300, China; 2023613022036@stu.zafu.edu.cn (C.Y.); jds@stu.zafu.edu.cn (D.J.); changlingwu@zafu.edu.cn (C.W.); 2 College of Agriculture and Biotechnology, Zhejiang University, Hangzhou 310058, China; jialichris1006@gmail.com (J.H.); 0619363@zju.edu.cn (Q.C.); 3 Zhejiang Zhiyi Biotechnology Co., Ltd., Hangzhou 325449, China; andyni2006@163.com; 4 Faculty of Chemistry, M.V. Lomonosov Moscow State University, 119991 Moscow, Russia; eremin_sergei@hotmail.com (S.A.E.); liliya106@mail.ru (L.I.M.); 5 School of Ecology and Environment, Xizang University, Lhasa 850012, China; gxf005@hotmail.com (X.G.); dekiy@utibet.edu.cn (J.D.)

**Keywords:** mycotoxin biotransformation, zearalenone biocontrol, yeast, glycosylation, stress resistance, enzyme activities

## Abstract

Zearalenone (ZEN) poses serious risks to human and animal health. Compared with physical and chemical methods, microbial transformation offers a safer and more sustainable strategy for ZEN detoxification. The yeast *Hannaella zeae*, isolated from the Qinghai–Tibet Plateau, showed the highest ZEN removal efficiency among 11 strains, achieving an 85.87% transformation rate within 36 h. Optimal conditions for ZEN transformation were determined by varying culture time, temperature, and pH. The products were putatively identified as zearalenone-14-β-D-glucopyranoside (C_24_H_32_O_10_) and zearalenone-16-β-D-glucopyranoside (C_24_H_32_O_10_) by UHPLC-Q-Orbitrap-HRMS. The safety of the mixed culture medium extract was further evaluated using a *Caenorhabditis elegans* model, showing significantly lower toxicity than untreated ZEN. *H. zeae* maintained high transformation efficiency under low temperature (57.48%) and acidic stress (47.10%), supported by active antioxidant enzymes (SOD, CAT, APX, GPx) and stress metabolites (trehalose, proline). Overall, this study identifies *H. zeae* as a promising, stress-tolerant biocontrol agent and elucidates its glycosylation-based detoxification mechanism, providing a foundation for future application in real food and feed systems.

## 1. Introduction

Zearalenone (ZEN), a potent estrogenic mycotoxin produced by *Fusarium* species (e.g., *Fusarium graminearum*, *Fusarium culmorum*), poses a significant threat to global agricultural systems and food safety [1,2]. As a non-steroidal endocrine disruptor, ZEN persists through food processing, contaminating cereals, nuts, dried fruits, and coffee beans [3], leading to acute poisoning (e.g., immune suppression) or chronic effects (e.g., carcinogenesis) via binding to estrogen receptors (ERα/β) [4,5]. To mitigate ZEN contamination, many control methods have been explored, such as physical, chemical [6], combined physicochemical methods, and microbial ways [7,8]. Physical methods include solvent extraction, irradiation, thermal treatments, cold plasma, catalytic processes, and biomass-based adsorption [9,10]. Among these, cell wall adsorption represents a typical physical detoxification mechanism. It relies on electrostatic interactions, hydrophobic bonds, or hydrogen bonding between cell wall components (such as β-glucan and mannan) and ZEN molecules to achieve ZEN removal, without involving enzymatic digestion or metabolic transformation processes. The *Sugiyamaella novakii* NCAIM Y.00986 eliminates 80% of ZEN through cell wall absorption [11]. Similarly, Pleurotus ostreatus can adsorb approximately 23% of ZEN under simulated digestive conditions [12]. Chemical strategies encompass ammoniation, alkaline hydrolysis, peroxidation, ozonation, and sulfite treatments [13]. It is noteworthy that innovations in materials science, such as the optimization of compound properties and functions through cocrystal technology [e.g., enhanced antifungal efficacy], also provide insights for developing novel control agents [14]. Although they can reduce ZEN contamination effectively, they also adversely impact food nutrient profiles leading to nutritional loss [15] and harmful compounds formation [16,17].

In contrast, microbial biodegradation is considered to have greater application potential due to its high safety and minimal impact on food quality [18]. Previous studies indicated that *Bacillus velezensis* strain ANSB01E degraded ZEN effectively through chitinases, carboxylesterases, and lactone hydrolases [19]. The recombinant laccase derived from Aspergillus oryzae and expressed heterologously in Pichia pastoris was reported to transform ZEN into 15-hydroxy-zearalenone [20]. Additionally, yeast from different sources has demonstrated exceptional detoxification capabilities. For example, *Rhodotorula dairenensis* can remove 98% of ZEN [21], while *Candida parapsilosis* ATCC 7330 can convert ZEN into the less toxic β-Zearalenol and ZEN-14,16-diglucoside [22]. However, yeast-mediated bioconversion also has stability issues. The detoxification efficacy of conventional yeast strains is highly dependent on their intracellular enzymatic activity and membrane integrity. However, these strains frequently encounter multiple environmental stressors in practical scenarios: low temperatures significantly retard metabolic rates and diminish the catalytic efficiency of key transforming enzymes; acidic environments not only directly inhibit enzymatic activity but also disrupt cellular energy metabolism [23]. Furthermore, composite stresses such as osmotic fluctuations and nutrient competition further compromise their physiological activity and survival rate. Collectively, these factors lead to a sharp decline in the ZEN transformation efficiency of existing yeasts under non-optimal conditions, constituting a major bottleneck for their industrial application. In this study, *H. zeae* was evaluated as a single functional strain to determine its independent transformation capacity and physiological mechanisms. However, in practical food and feed environments, microbial processes are often more stable when using synthetic consortia or complementary strains. Different yeasts contribute distinct advantages, such as superior acid tolerance, increased low-temperature activity, or higher catalytic efficiency [24]. Therefore, future studies could explore constructing multi-strain synergistic systems in which complementary metabolic strengths are combined and optimized for different storage temperatures, pH conditions, and substrate compositions. Especially under environmental stress conditions, its efficiency is significantly limited; this has become the main bottleneck in its practical application.

Currently, in research on the yeast-mediated degradation of ZEN, the mechanisms of conversion under stress conditions and the selection of highly efficient strains have become key areas of focus. Through strategies such as directed evolution, stress adaptation domestication, and molecular modification, researchers are working to enhance the yeast conversion efficiency of ZEN under adverse conditions. Notably, screening indigenous microorganisms with high degradation capabilities from high-altitude, extreme natural environments, or heavily polluted habitats has become an essential approach for obtaining strains with strong tolerance and prominent activity. There is empirical evidence that crops on the Tibetan Plateau are also affected by *Fusarium* mycotoxins. A recent survey of freshly harvested highland barley (qingke) from Tibet detected *Fusarium* mycotoxins, including zearalenone (ZEN), with a detection rate of 6%. Some samples even exceeded the Chinese maximum permitted level of 60 μg/kg for ZEN in food, indicating potential food safety risks during harvest and storage [25]. Earlier regional investigations and comprehensive risk assessments have also pointed out that cereals grown on the Tibetan Plateau may experience high levels of mycotoxin exposure under certain environmental and storage conditions, further supporting the practical significance of screening stress-tolerant detoxifying microorganisms native to this region [26]. The Tibet region, with its unique high-altitude ecological environment—including low temperatures, low oxygen levels, strong ultraviolet radiation, and relatively pristine land use practices—harbors rich and distinctive microbial resources. These microorganisms may have evolved exceptional metabolic potential and environmental adaptability to extreme conditions. Therefore, screening yeast strains with high-efficiency ZEN degradation capabilities from various environments in Tibet not only increases the likelihood of success but also provides valuable insights into elucidating the biological pathways for efficient ZEN degradation and developing novel stress-tolerant degradation agents. Although numerous ZEN-degrading bacteria and yeasts have been reported in the past decade, most strains exhibit significant declines in degradation efficiency under environmental stresses such as low temperature, acidity, or oxidative pressure during real food or feed applications. This instability has become a major bottleneck restricting the industrialization and field application of microbial detoxification. In contrast, strains isolated from extreme habitats such as the Tibetan Plateau may have undergone long-term natural stress selection, resulting in enhanced stress tolerance and metabolic stability. However, systematic studies simultaneously investigating ZEN transformation efficiency, biochemical detoxification pathways, and environmental tolerance of such extremophilic yeasts remain scarce. The remarkable adaptability of yeast to environmental stresses is largely rooted in the structural complexity and functional diversity of its regulatory proteins, enabling rapid metabolic reprogramming in response to fluctuating conditions [27,28]. Therefore, this study screened a novel Tibetan yeast strain and comprehensively evaluated its ZEN biotransformation mechanism, metabolite safety, and stress resistance, aiming to provide microbial candidates suitable for practical food and feed applications. In addition to tolerance, glycosylation is considered an essential pathway for yeast degradation of ZEN at the metabolic mechanism level.

In the microbial detoxification of ZEN, glycosylation is considered one of the key biotransformation pathways. This process involves introducing glucose groups into the hydroxyl sites of ZEN molecules to form glycosidic bonds, thereby modifying or masking their active sites [26]. Glycosylation usually involves the enzymatic conjugation of glucose moieties to hydroxyl groups on the ZEN, forming glycosidically linked derivatives. For example, it has been reported that *Candida parapsilosis* ATCC 7330 can convert ZEN into ZEN-14,16-diglucoside. This finding not only verifies the role of yeast in glycosylation modification, but also provides experimental evidence for revealing its potential detoxification mechanism [21]. As a result, screening stress-tolerant yeast from Tibet is a feasible method to solve the stability issue of biotransformation with microorganisms. Based on this premise, this study aimed to: (1) screen and identify a potent ZEN-transforming yeast from diverse environments in the Tibet region; (2) comprehensively characterize its biotransformation efficiency, elucidate the detoxification pathway through metabolite identification, and evaluate product safety; and (3) assess the strain’s stress tolerance to low temperature and acidity, investigating the physiological mechanisms ensuring its stable performance.

## 2. Results and Discussion

### 2.1. ZEN Biotransformation Yeast Isolation and Identification

**Figure 1:**
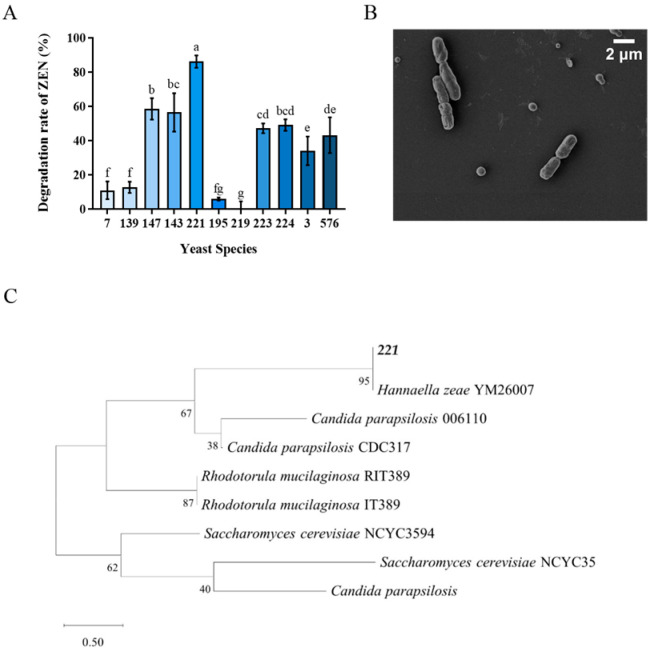
Isolation and identification of Tibetan yeast strains. (**A**) The rate at which different yeast strains transform ZEN within the Nutrient Yeast Dextrose Broth (NYDB) medium. The data presented in columns marked with distinct letters indicate statistically significant differences, as determined via Duncan’s New Multiple Range Test at a significance level of *p* < 0.05 (*n* = 3); (**B**) Morphological characterization via Scanning Electron Microscopy of *H. zeae*. (**C**) A phylogenetic tree analysis of strain 221 was conducted using a partial 26S rDNA D1/D2 domain sequence and the neighbor-joining algorithm. The percentages indicated above the branches represent the confidence limits expressed as percentages (the estimates were determined via bootstrap analyses involving 1000 replicates).

The results of eleven yeast strains transformation effect on ZEN were shown in Figure 1A. Among them, Tibetan yeast strains No. 7, 139, 195 and 219 exhibited inferior efficiency, whose, transformation rates were lower than 20%. The transformation rates of Tibetan yeast strains No. 223, 224, 3 and 576 were lower than 50%. While the No. 147, 143 and 221 showed better transformation rates higher than 60%. The yeast strain No. 221 showed the highest ZEN transformation capacity with a transformation rate of 86.17%. So, it was selected as a candidate for further study. Notably, the superior performance of No. 221 highlights the significance of exploring extremophilic microorganisms from unique habitats such as Tibet, since environmental pressures in these regions may have driven the evolution of enhanced detoxification capabilities [28]. The phylogenetic analysis confirmed that the taxonomic identity of the No. 211 yeast strain had a 99.9% similarity with *Hannaella zeae*, which was shown by a neighbor-joining phylogenetic tree (Figure 1C). Meanwhile, the scanning electron microscopy (SEM) revealed its shape was rod-shaped with featureless surfaces and intact cellular envelopes (Figure 1B).

### 2.2. Biotransformation Effect of Tibetan Yeast on ZEN

**Figure 2:**
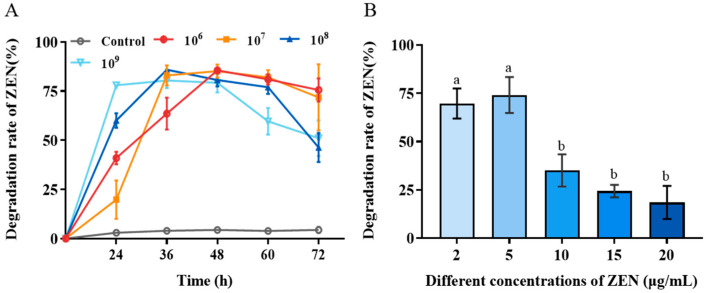
Biotransformation effect of *H. zeae* on ZEN. (**A**) The transformation effects of different yeast concentrations. After using different treatment methods, different states of yeast cell suspension were selected to add ZEN at an initial concentration of 5 μg/mL; (**B**) The effect of initial ZEN concentration on the transformation of zearalenone (ZEN) by *H. zeae*. The data presented in columns marked with distinct letters indicate statistically significant differences, as determined via Duncan’s New Multiple Range Test at a significance level of *p* < 0.05 (*n* = 3).

The biotransformation performance of *H. zeae* at four different concentrations (1 × 10^6^, 1 × 10^7^, 1 × 10^8^ and 1 × 10^9^ cells/mL) was systematically investigated to identify optimal biotransformation parameters. The results revealed that *H. zeae* exhibits a clear concentration-dependent pattern in the biotransformation of ZEN, with both excessively low and high inoculation concentrations leading to suboptimal transformation efficiency (Figure 2A). At the lowest cell concentration (1 × 10^6^ cells/mL), ZEN removal gradually accelerated, reaching a transformation efficiency of 63.52% at 36 h. The intermediate concentration (1 × 10^7^ cells/mL) displayed a progressive increase in transformation efficiency, peaking at 48 h with 85.13%. Lower cell concentration (1 × 10^6^ and 1 × 10^7^ cells/mL) experienced extended adaptation phases, correlating with delayed transformation initiation. This finding is consistent with previous results. A systematic investigation of the inoculation proportion of the *Bacillus* strain X13 and the initial substrate concentration on ZEN degradation efficiency revealed an optimal inoculation level and a pattern of efficiency decline due to high substrate changes [29]. At higher cell concentration (1 × 10^8^ cells/mL), an accelerated transformation rate was observed, reaching peak efficiency at 36 h, followed by a decline to baseline levels at 72 h with 46.27%. The highest cell concentration (1 × 10^9^ cells/mL) peaked rapidly at 24 h with 77.95%. It has resulted in premature carbon/nitrogen resource depletion and reduced viable biomass. In microbial detoxification systems, a low inoculation density may result in insufficient biomass, thereby limiting the overall rate of the enzymatic reaction; conversely, an excessively high inoculation density can trigger nutrient competition and the accumulation of metabolic by-products, which may subsequently suppress cellular activity and the stability of transforming enzymes [30]. Therefore, the concentration of 1 × 10^8^ cells/mL was selected as an effective and suitable concentration for the next study. This concentration maintains high conversion efficiency while also maintaining metabolic activity for a long period of time.

The effect of initial ZEN concentration on *H. zeae* biotransformation was presented in Figure 2B. With the initial ZEN concentration increased, the transformation efficiency significantly decreased. When the initial ZEN concentration was enhanced from 10 to 20 μg/mL, the biotransformation rate was reduced from 75% to less than 25%. This suggests that high ZEN concentrations was likely to exert toxic effects on yeast cells, potentially inhibiting the activity of key transforming enzymes or inducing cell membrane damage, ultimately leading to reduced transformation efficiency [31].

### 2.3. Inhibition of H. Zeae to Fusarium Graminearum Growth

**Figure 3:**
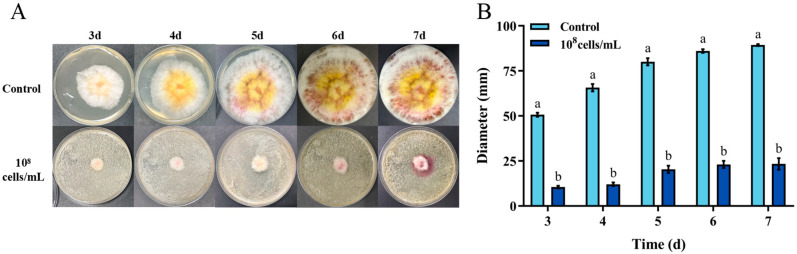
In vitro inhibition of *Fusarium graminearum* growth by *H. zeae*. (**A**) Colony morphology of *F. graminearum* on PDA; (**B**) Colony diameter of *F. graminearum* after 3–7 d of growth on PDA. Inhibition of *F. graminearum* growth by *H. zeae* at a concentration of 1 × 10^8^ cells/mL. The data represent the mean ± SD (*n* = 3) at each time point. The data presented in columns marked with distinct letters indicate statistically significant differences, as determined via Duncan’s New Multiple Range Test at a significance level of *p* < 0.05 (*n* = 3).

The *Fusarium graminearum* is a primary producer of ZEN. The biocontrol effect of *H. zeae* against *F. graminearum* is demonstrated in Figure 3. After 7 days incubation, *H. zeae* significantly inhibited fungal colony growth compared to the control at the concentration of 1 × 10^8^ cells/mL. The fungal colony diameter of control was 89.33 mm, while the one of treatment was 23.33 mm. Yeasts typically inhibit *Fusarium graminearum* through multiple mechanisms, including nutrient and spatial competition, production of volatile metabolites, suppression of spore germination, secretion of siderophores, and release of extracellular lytic enzymes such as chitinases and β-1,3-glucanases. Notably, nutrient competition has been identified as a primary antagonistic mechanism of yeast against *F. graminearum* [32]. Yeast strains such as *Kluyveromyces marxianus*, *Meyerozyma caribbica*, and *Wickerhamomyces anomalus* effectively inhibit the growth of grain-associated *Fusarium* in vitro and maize seeds through the antagonistic mechanism of nutrient competition [33].

### 2.4. Transformation Effect of Different Yeast Cell Treatments

**Figure 4:**
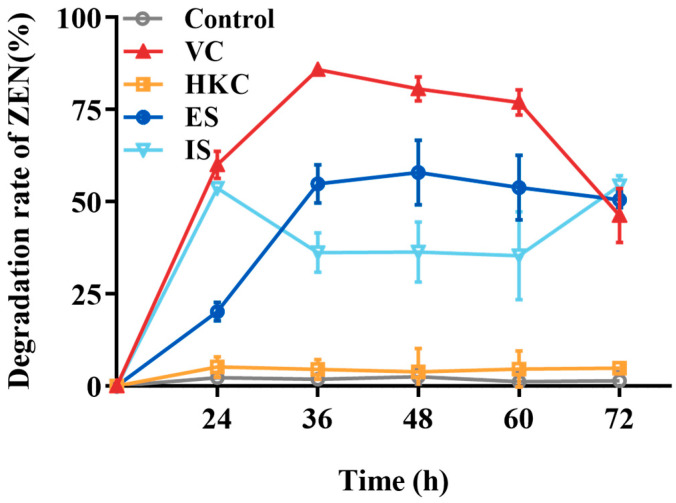
The ZEN transformation effects of different *H. zeae* treatments. VC stands for viable cells; HKC stands for heat-inactivated cells; ES stands for extracellular supernatant; IS stands for intracellular substances.

The ZEN transformation capabilities of different treated *H. zeae* cell solutions were shown in Figure 4. The heat-killed cells exhibited negligible transformation activity, which was less than 5%. Both extracellular and intracellular substances demonstrated partial transformation effects with 54.87% and 36.18% transformation rates after 36 h incubation, respectively, the highest transformation capacity belonged to the *H. zeae* viable cells peaking at 85.87%. According to the results above, the detoxification mechanism was mainly elucidated as intracellular and extracellular enzymatic transformation. The enzymatic transformation plays a crucial role, wherein specific enzymes cleave the lactone ring structure of ZEN, thereby effectively neutralizing its estrogenic activity [34]. For instance, *Saccharomyces cerevisiae* (*S. cerevisiae*) secretes lactonase enzymes capable of hydrolyzing the lactone group within ZEN’s lactone ring [35]. Likewise, the ZEN transformation efficiency of *Bacillus velezensis* L9 extracellular supernatant treated with protein denaturants (e.g., sodium dodecyl sulfate and protease K) decreased significantly from 54.87 to 12% [17], further confirming the key role of transforming enzymes in this process. Beyond direct enzymatic cleavage of ZEN, detoxification also involves activation of cellular antioxidant defenses. ZEN exposure generates excessive reactive oxygen species (ROS), causing oxidative damage to cellular macromolecules, as indicated by malondialdehyde (MDA) accumulation. To mitigate this stress, yeast cells upregulate antioxidative enzymes, includingsuperoxide dismutase (SOD), catalase (CAT), and glutathione s-transferase (GST), while glutathione peroxidase (GPx) activity decreases, as observed in *Schizosaccharomyces pombe* [36]. These changes suggest that redox homeostasis works synergistically with ZEN-degrading enzymes to enhance yeast survival and detoxification efficiency.

### 2.5. Effect of Cycloheximide on ZEN Transformation

**Figure 5:**
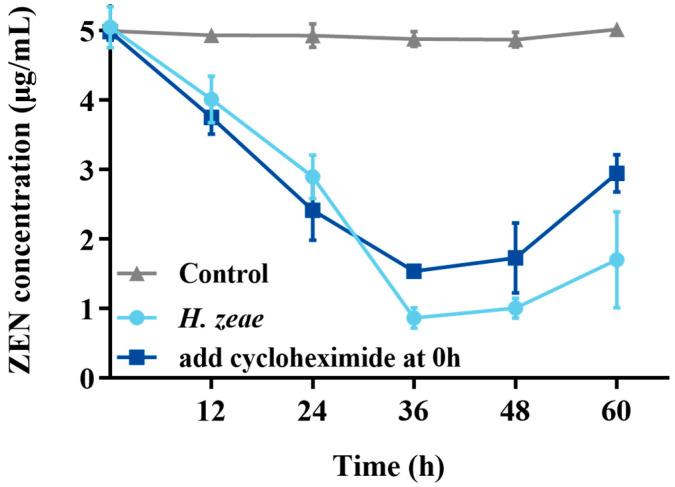
Effect of cycloheximide on the ZEN transformation. The *x*-axis represents the time of incubation and the *y*-axis represents the concentration of ZEN in the medium. The data represent the mean ± SD (*n* = 3) at each time point.

In order to verify the intracellular substances action on transformation, cycloheximide inhibition test was conducted. The results showed that control (without cycloheximide) had significantly transformation effect than treatment (Figure 5). Remarkably, the residual ZEN concentration at 36 h in the experimental group supplemented with cycloheximide at the initial incubation stage (1.54 μg/mL) was significantly higher, approximately two times greater than that observed in the group without cycloheximide (0.77 μg/mL). These findings suggest that although cycloheximide partially inhibited ZEN transformation mediated by *H. zeae*, the inhibitory effect was limited. These observations are consistent with previous reports indicating that cycloheximide delays mycotoxin transformation in yeast-based systems. For instance, a similar inhibitory pattern was observed in the transformation of patulin by *Pichia caribbica*, where cycloheximide addition significantly delayed toxin removal [15]. Mechanistically, cycloheximide inhibits intracellular enzyme synthesis; therefore, the residual transformation capacity (ZEN concentration reduced to 1.54 μg/mL) strongly suggests that *H. zeae* predominantly relies on intracellular synthesized enzymes. This further underscores that yeast intracellular substances are the key drivers of ZEN biotransformation. As a protein synthesis inhibitor, cycloheximide not only halts the production of new enzymes but may also impact overall cellular metabolic activity and survival status. Although cell viability was not directly measured in this experiment, the residual transformation activity suggests that some pre-existing enzymes retained partial function, and/or the cells maintained a degree of metabolic capacity under inhibitor stress. Future studies should incorporate direct viability assessments to more comprehensively evaluate the physiological impact of cycloheximide on yeast cells.

### 2.6. Transformation Product Analysis and Safety Evaluation

#### 2.6.1. Transformation Product Analysis

**Figure 6:**
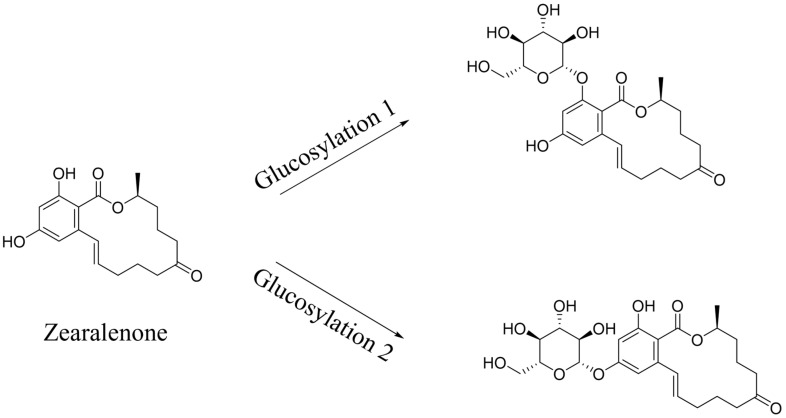
Metabolic transformation products.

**Figure A1:**
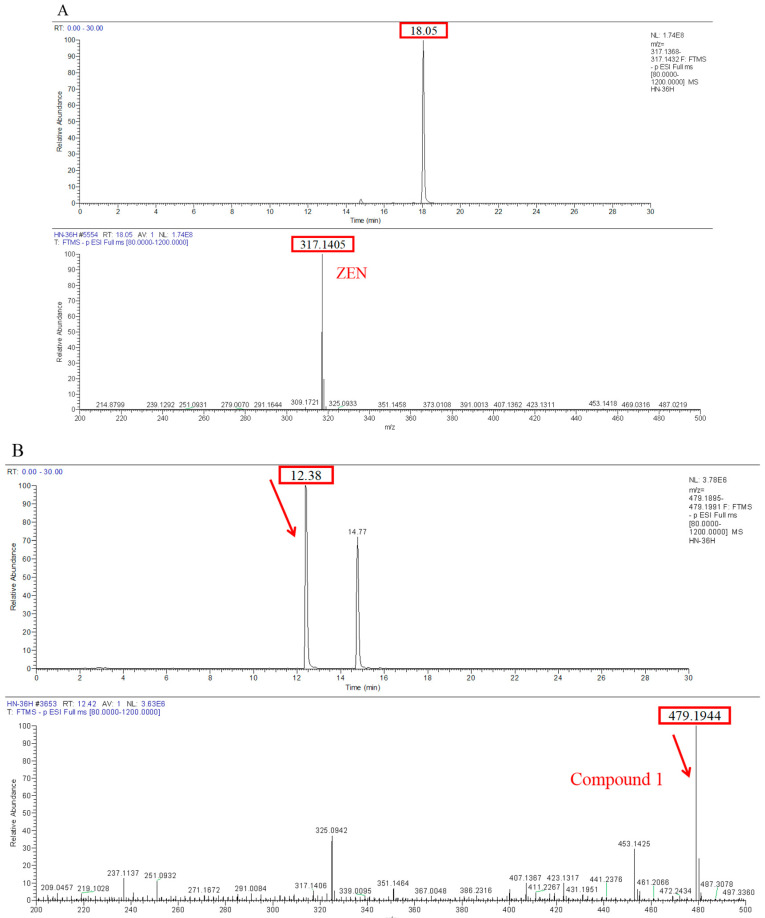

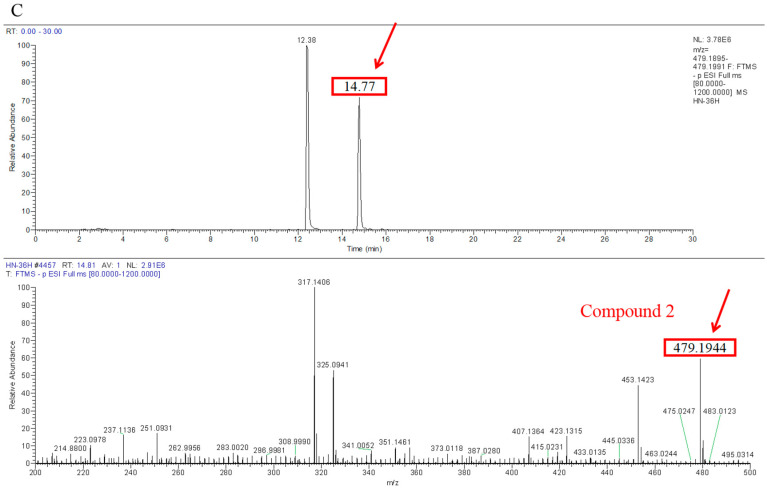
Comparison of ultra-high performance liquid chromatography quadrupole-orbitrap high-resolution mass spectrometry (UHPLC-Q-Orbitrap-HRMS) analysis results of ZEN and transformation products. (**A**) ZEN; (**B**) Compound 1; (**C**) Compound 2.

Transformation products of ZEN by *H. zeae* were systematically analyzed using ultra-high performance liquid chromatography quadrupole-orbitrap high-resolution mass spectrometry (UHPLC-Q-Orbitrap-HRMS). Chromatographic analysis (Figure 6) revealed the parent ZEN compound at a retention time of 18.05 min, with its deprotonated molecular ion [M-H]^−^ *m*/*z* 317.1368 (Figure A1). Two novel metabolites were identified after 36 h co-cultivation of *H. zeae* with ZEN: Compound 1 (retention time: 12.38 min) and Compound 2 (retention time: 14.77 min), both exhibiting [M-H]^−^ *m*/*z* 479.1944. The molecular mass difference between these metabolites and ZEN was 162.0576 Da, consistent with the theoretical mass increment for monoglycosylation. This supports the proposed molecular formula C_24_H_32_O_10_, characteristic of monoglucosylated conjugates of ZEN. The transformation aligns with glycosyltransferase- catalyzed mechanisms. As reported [21], the ZEN-glucoside (*m*/*z* 481.2068 [M + H]^+^) generated by *Candida parapsilosis* ATCC 7330 matches the molecular mass of the present products. Additionally, studies of *Cunninghamella echinulate* confirmed the generation of zearalenone-14-β-D-glucopyranoside (ZEN-14-G) and zearalenone-16-β-D-glucopyranoside (ZEN-16-G) (*m*/*z* 481.2079 [M-H]^−^) [37]. Cross-referencing mycotoxin databases suggests that Product 1 and 2 correspond to ZEN-14-G and ZEN-16-G, respectively. Previous studies have identified specific fungal strains capable of mediating this transformation. These findings demonstrate that glucose conjugation is also a biologically significant pathway for microbial ZEN detoxification apart from enzyme degradation. The results indicate that *H. zeae* mediates ZEN biotransformation by glycosyltransferase-catalyzed glucosylation, yielding two stereoisomers. This observation conforms to established fungal glycosylation detoxification models, though definitive configuration requires NMR validation. The glycosylated products of ZEN (ZEN-14-G and ZEN-16-G) identified in this study are consistent with the derivatives reported in other microbial systems, such as *Candida parapsilosis* ATCC 7330 and *Cunninghamella echinulate* [21]. This consistency underscores glycosylation as a conserved detoxification strategy shared across diverse microorganisms. Nevertheless, the high transformation efficiency exhibited by *H. zeae* under low temperature and acidic conditions provides it with a distinct advantage for practical applications in non-ideal environments. Although glycosylation substantially diminishes the estrogenic activity of ZEN in vitro [38], this structural modification exhibits potential reversibility in the gastrointestinal physiological environment, which may lead to the re-release of the parent ZEN compound. Therefore, the ZEN glucosylated derivatives identified in this study should be defined as temporarily detoxified forms rather than permanently inactivated products. To fully validate their safety in practical application scenarios, subsequent systematic stability evaluations and bioavailability tests will be conducted using simulated gastrointestinal digestion models in next test. The intricate metabolic potential of microorganisms often requires an integrated computational and experimental approach to fully elucidate detoxification mechanisms. Supporting this notion, Liu et al. employed genomics and molecular docking techniques to reveal the interaction patterns between microbial enzymes and ZEN, thereby providing novel insights for the rational design of high-efficiency detoxifying strains [39].

Notably, previous toxicological research has demonstrated that glycosylated ZEN metabolites (such as ZEN-14-G and ZEN-16-G) exhibit significantly reduced estrogenic toxicity compared with the parent compound. Published studies have confirmed that these metabolites show markedly lower biological toxicity in both in vivo and in vitro models [40,41]. Therefore, the glycosylation observed in this study is consistent not only with established fungal detoxification pathways but also with the detoxification trend reported in the existing toxicological literature. However, it should be noted that glycosylated derivatives may undergo partial hydrolysis in the gastrointestinal tract, potentially releasing the parent compound ZEN again. Future work will therefore focus on studying purified individual metabolites and assessing their metabolic stability and bioavailability using simulated gastrointestinal digestion models.

#### 2.6.2. Product Safety Evaluation

**Figure 7:**
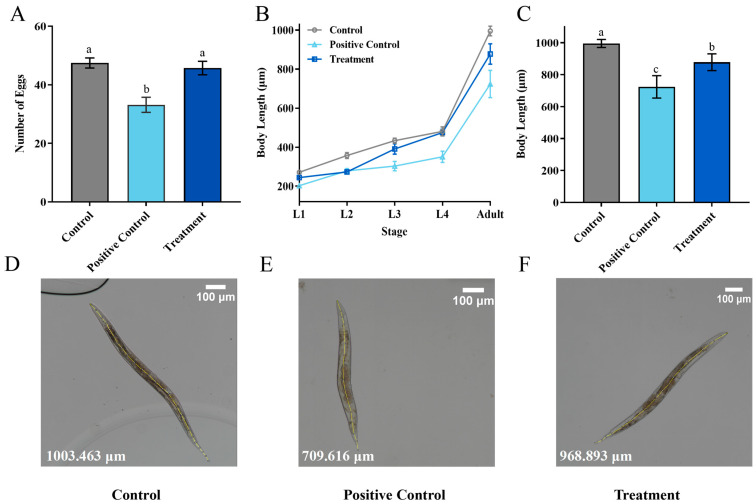
Transformation Product Safety Evaluation. (**A**) The oviposition of *Caenorhabditis elegans (C. elegans)* after exposure to different concentrations of ZEN; (**B**) the effects of different concentrations of ZEN on *C. elegans* body length; (**C**) the body length of *C. elegans* at each stage; (**D**–**F**) Detailed diagram of body length measurement. The ZEN concentration was 0 μg/mL in the control group, 5 μg/mL in the positive control group, and 0.7 μg/mL in the treatment group. The data presented in columns marked with distinct letters indicate statistically significant differences, as determined via Duncan’s New Multiple Range Test at a significance level of *p* < 0.05 (*n* = 10).

To study the potential toxic effects of ZEN on *Caenorhabditis elegans* (*C. elegans*), the quantity of offspring was analyzed in N2 wild-type *C. elegans*. The results showed that the positive control (5 μg/mL ZEN) significantly reduced the average offspring quantity of *C. elegans* after 72 h compared with control, from 47.4 to 33.2 (Figure 7A). In contrast, the offspring quantity of treatment with a residual ZEN concentration at 0.7 μg/mL was 45.7, showing no significant difference compared with the healthy nematode (control), which indicates that the biotransformation of *H. zeae* was able to remove the toxicity of ZEN. This result is consistent with the findings of other researchers. The study demonstrated that at concentrations of 7.5 μg/mL and 37.5 μg/mL, ZEN reduced the number of offspring to 39.0 and 31.7, respectively, showing a dose-dependent pattern [42].

The growth of *C. elegans* is commonly assessed by body length measurement. As depicted in Figure 7, both the positive control and treatment groups showed developmental impairment. From larval L1 to the adult stage, the growth of treatment was delayed significantly (Figure 7B). The body length of adults in the positive control group during the adult stage was reduced to 271.98 μm (Figure 7C). Body length differences were minimal during early developmental stages. For instance, at the L4 larval stage, the body length of the treatment group was 474.80 μm, slightly shorter than that of the control group, which measured 480.77 μm. At the adult stage, the treatment group exhibited an average body length of 877.84 μm. Compared to the positive control (5 μg/mL ZEN), the treatment group containing the medium after *H. zeae* transformation (residual ZEN at 0.7 μg/mL) led to an approximately 37.7% recovery in offspring quantity and a 22.3% increase in body length at the adult stage. Based on the reversal of these key toxicity endpoints, the overall toxic effect of the transformed medium was reduced by more than 60% compared to the pure ZEN control, demonstrating a significant detoxification effect. This value was longer than that of the positive control group at the same developmental stage, yet remained lower than the corresponding measurement observed in the untreated control group. These findings suggest that the treatment may partially mitigate growth inhibition while not fully restoring normal developmental parameters. These findings suggest that residual ZEN (0.7 μg/mL) after transformation by *H. zeae* exerts a slight yet measurable effect on *C. elegans* body length. This concentration-dependent growth inhibition aligns with previous observations. For instance, systematic comparisons among major foodborne mycotoxins (e.g., Aflatoxin B1, Deoxynivalenol, Fumonisin B1, T-2 toxin, and ZEN) revealed reproductive toxicity induced by ZEN comparable to that of AFB_1_, though effects differed concerning lethality, body length, and lifespan in C. elegans. Furthermore, exposure to ZEN under conditions influenced the *C. elegans* wild-type N2 strain [43]. This finding is supported by a growing body of evidence highlighting the pivotal role of microbial metabolites in host physiology. Specifically, some researchers demonstrated that alterations in microbial metabolites, such as a reduction in phytosphingosine, can significantly impair host physiological functions. The similarity between the growth and developmental damage induced by ZEN in our study and the functional impairments linked to microbial metabolite shifts reported by Li et al. reinforces the concept that perturbations in microbial-derived molecules are a critical mechanism underlying toxin-mediated toxicity [44].

Owing to the complexity of the tested extract, which included both residual ZEN and microbial biotransformation products, the present findings solely indicate a detoxification trend for the entire system. The definitive toxicological profiling of specific metabolites, ZEN-14-G and ZEN-16-G, will be isolated and assessed individually in further study. It should be emphasized that the present toxicity evaluation was based on extracts containing residual ZEN together with its glycosylated metabolites (ZEN-14-G and ZEN-16-G). Previous studies have demonstrated that these glycosylated derivatives possess substantially lower estrogenic toxicity than the parent toxin [45]. Our experimental results are consistent with these findings. Nevertheless, the possibility that gastrointestinal enzyme hydrolysis may lead to partial deconjugation and reactivation of ZEN cannot be excluded. Therefore, subsequent research will focus on independent toxicity assessments of purified metabolites and evaluation of their stability and safety under simulated gastrointestinal conditions.

### 2.7. Tolerance of Tibetan Yeast to Acid and Temperature Stress

**Figure 8:**
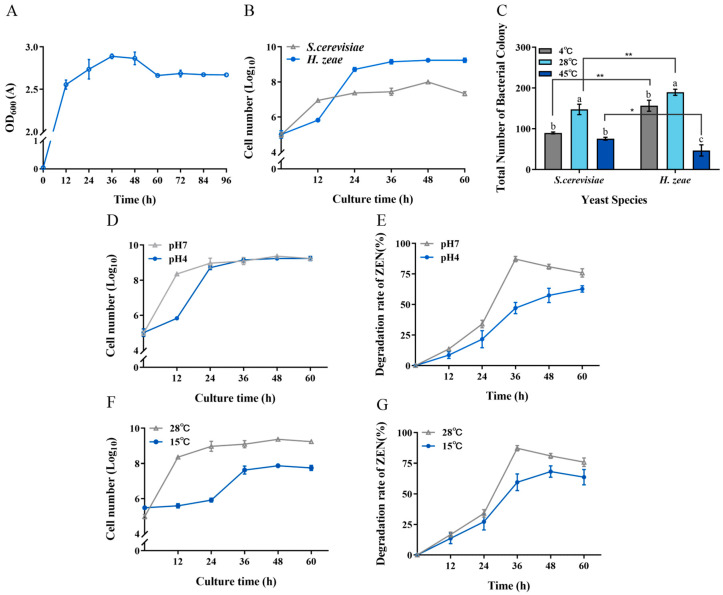
Tolerance of *H. zeae* to temperature and acidic stress. (**A**) The growth curve of strain *H. zeae*; (**B**) The growth of *H. zeae* and laboratory *Saccharomyces cerevisiae* (*S. cerevisiae*) at pH 4 The viability of yeasts was determined via dilution plating on NYDA plates; (**C**) The growth of *H. zeae* at different temperatures. * indicates *p* < 0.05 (statistically significant difference), and ** indicates *p* < 0.01 (highly statistically significant difference). (**D**) Growth curve under acidic stress conditions; (**E**) The transformation rate of ZEN under acidic stress conditions; (**F**) Growth curve under low temperature stress conditions; (**G**) The transformation rate of ZEN under low temperature stress conditions. The viability of yeasts was determined via dilution plating on NYDA plates. The data represent the mean ± SD (*n* = 3) at each time point. The data presented in columns marked with distinct letters indicate statistically significant differences, as determined via Duncan’s New Multiple Range Test at a significance level of *p* < 0.05 (*n* = 3).

The growth characteristics of the isolated yeast strain, *H. zeae*, were evaluated under various temperature and pH conditions. Initially, the growth curve of the strain was established (Figure 8A). The first stage of cellular growth occurred from approximately 1 to 12 h. Subsequently, cells entered the logarithmic growth phase, characterized by rapid proliferation and maximum division rates. After approximately 12 h, cell proliferation slowed considerably, transitioning into the stationary phase. This decline in growth rate corresponded to nutrient depletion and accumulation of metabolic byproducts, resulting in stabilization of cell density at a relatively constant peak level.

To further characterize the biological properties of *H. zeae*, its tolerance to acidic stress was compared with that of the standard yeast strain *S. cerevisiae* in an acidic NYDB medium at pH 4 (Figure 8B). Notably, during the initial 18 h incubation period, *S. cerevisiae* exhibited superior acid tolerance compared to *H. zeae*. However, after 18 h of incubation, *H. zeae* cell counts significantly surpassed those of *S. cerevisiae*, rapidly increasing and stabilizing at approximately 1.62 × 10^9^ CFU/mL. This observation indicates that *H. zeae* possesses remarkable acid tolerance, suggesting its potential for rapid activation, growth, and proliferation in highly acidic environments, such as human or animal gastric fluids. Previous research supports this observation, indicating microbial adaptation mechanisms under acidic stress. For instance, it has been reported that *Bacillus cereus* BC7 could effectively remove ZEN (89.31%) from Luria–Bertani medium under simulated gastric fluid conditions at 37 °C after 24 h [46]. Moreover, *S. cerevisiae* reportedly responds to acetic acid stress through structural modifications and increased rigidity of the cell wall [23]. Future research will focus on evaluating the capability of *H. zeae* to transform ZEN under simulated gastric fluid conditions and determining whether structural changes in cellular organelles contribute to acid stress tolerance. The stress tolerance and efficient detoxification capability of *H. zeae* are likely rooted in its unique genomic background. The potential for further enhancing these traits is supported by recent advances in yeast genomic editing. For instance, Sohail demonstrated that targeted reinforcement of stress-responsive genes significantly improved the survival rate and metabolic activity of yeast under highly acidic conditions. This provides a compelling rationale and a methodological framework for the future engineering of *H. zeae* to further augment its detoxification potential [47].

Regarding temperature tolerance, the low temperature resistance of *H. zeae* was similar to that of *S. cerevisiae* (Figure 8C). Interestingly, *H. zeae* exhibited comparatively lower tolerance to high-temperature stress than S. cerevisiae. This difference aligns with findings from previous studies [48], demonstrating that although S. cerevisiae has relatively high thermotolerance and recovery capacity, other yeast strains may lack comparable resilience under similar stress conditions. Under acidic conditions (pH 4), *H. zeae* exhibited a ZEN transformation rate of 47.10% after incubation (Figure 8E). Notably, this strain demonstrated significantly enhanced tolerance to low temperature stress, achieving 57.48% transformation under suboptimal conditions. (Figure 8G). These results underscore the variability in thermal adaptability among different yeast species, reflecting distinct growth patterns and stress responses under elevated temperatures. Furthermore, at the optimal temperature of 28 °C, *H. zeae* showed superior growth and colony proliferation compared to *S. cerevisiae*. At low temperatures (4 °C), *H. zeae* demonstrated significantly enhanced cold tolerance relative to S. cerevisiae, highlighting its substantial psychrotolerant properties. Given that current research on psychrotolerant yeast strains remains limited, the robust performance of *H. zeae* at low temperature and acidic conditions presents a distinct advantage for its potential application in biotechnological fields. The findings from this study therefore not only confirm the strain’s high activity under environmental stress but also provide valuable insights and novel research directions for future studies on stress-resistant microbial resources. The robust stress tolerance and efficient glycosylation-based detoxification observed in *H. zeae* likely originate from its unique genetic background, shaped by the extreme selective pressures of the Qinghai–Tibet Plateau. This premise is strongly supported by emerging genomic evidence from other extremophilic fungi. For instance, whole-genome sequencing of *Trametes sanguinea* ZHSJ, a strain derived from a distinctive ecological niche, revealed a substantial arsenal of genes encoding stress-responsive proteins and detoxification enzymes, such as cytochrome P450s and glycosyltransferases, which are postulated to underpin its exceptional adaptive capabilities [49]. This pattern suggests that extreme environments serve as invaluable reservoirs for microbial strains endowed with unique catabolic genes. Therefore, the remarkable environmental adaptability and biotransformation efficiency of our *H. zeae* isolate are not isolated phenomena but are consistent with a broader evolutionary strategy. It is highly plausible that *H. zeae* possesses a similarly unique genomic repertoire, particularly enriched in glycosyltransferases responsible for the observed ZEN glycosylation, a hypothesis that warrants confirmation by future genomic sequencing.

### 2.8. Related Substances Variation in Yeast to ZEN and Stress

#### 2.8.1. Determination of the Correlation Enzyme Activity

**Figure 9:**
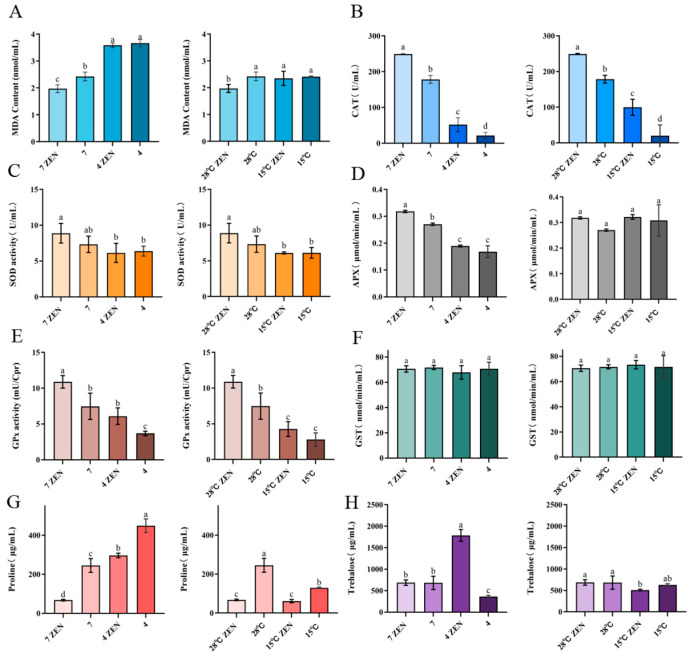
The Influence of ZEN on the generation of oxidative stress in *H. zeae*. (**A**) Malondialdehyde. (**B**) Catalase (CAT). (**C**) Superoxide dismutase. (**D**) Ascorbate peroxidase (APX). (**E**) Glutathione peroxidase (GPx). (**F**) Glutathione S-transferase (GST). (**G**) Proline. (**H**) Trehalose. The data presented in columns marked with distinct letters indicate statistically significant differences.

The primary end-product of lipid peroxidation, MDA, serves as a critical biomarker for evaluating oxidative membrane damage and integrity [50]. Under acidic conditions (pH 4), MDA content in ZEN-treated groups increased significantly to 1.7 times compared to the pH 7 controls, while untreated groups exhibited 1.5 times increase (Figure 9A). At 15 °C, MDA levels in ZEN-treated groups rose significantly to 1.2 times that of the 28 °C controls, whereas untreated groups showed no significant change (a difference of 0.01 μmol/min/mL). Notably, MDA accumulation at pH 4 was 1.5 times higher than that at 15 °C, indicating stronger oxidative membrane damage under acidic conditions.

The cells counteract oxidative stress through enzymatic defenses, including CAT, SOD, and ascorbate peroxidase (APX) [51]. Under acidic conditions (pH 4), ZEN-treated groups exhibited significantly reduced CAT (20.80%), SOD (69.13%), and APX (59.58%) activities compared to the pH 7 controls (Figure 9B–D). The untreated groups showed reductions to 12.13%, 87.12%, and 62.32%, respectively. At 15 °C, the ZEN-treated groups demonstrated significantly decreased CAT (40.04%) and SOD (68.75%) activities but unchanged APX (difference: 0.004 μmol/min/mL) compared to the 28 °C controls. Untreated groups had reduced CAT (11.25%) and SOD (83.37%) activities with stable APX activity (difference: 0.04 μmol/min/mL). The activities of CAT and APX in ZEN-treated groups at 15 °C were 1.9 times and 1.7 times higher than those at pH 4. SOD activity remained significantly higher in ZEN-treated groups than in untreated controls under both stress conditions. Acidic stress universally exerted stronger suppression on antioxidant enzymes than hypothermic stress. ZEN exposure exacerbated lipid peroxidation under both stresses while differentially modulating enzyme activities. These coordinated alterations in SOD, CAT, and APX activities demonstrate their pivotal roles in regulating radical homeostasis during combined stressors [52]. The significant decrease in APX and CAT activities under acidic conditions may be attributed to several interrelated mechanisms: low pH potentially induces alterations in enzyme protein conformation, protonation of active sites, and the accumulation of intracellular H_2_O_2_, which can lead to enzyme auto-inactivation. Furthermore, the oxidative stress induced by ZEN exposure may have synergistically exacerbated irreversible damage to these enzymes.

GPx plays a critical role in scavenging lipid peroxides and hydrogen peroxide (H_2_O_2_), effectively preventing cellular damage and maintaining intracellular redox balance [53]. GST participates in mycotoxin transformation through direct conjugation with toxins such as ZEN. Under varying pH conditions, ZEN exerted differential effects on GPx activity. At pH 4, GPx activity in ZEN-treated groups increased significantly by 39.50% (1.65 times) compared to the controls (Figure 9E). At pH 7, activity similarly rose by 31.35% (1.46 times). Furthermore, GPx activity in ZEN-treated groups at pH 4 was 1.43 times higher than in the ZEN-treated groups at 15 °C. These findings indicate a significant induction of GPx activity in *H. zeae* under 5 μg/mL ZEN induction. Collectively, *H. zeae* activates GPx to eliminate ROS induced by mycotoxins under multi-stress conditions, demonstrating robust self-protection and ability to maintain redox homeostasis.

GST activity in *H. zeae* did not show any significant alteration under ZEN stress (Figure 9F). This suggests that GST remained inactivated during oxidative stress, which indicates that its activation was potentially suppressed at 5 μg/mL ZEN and thus precluded its involvement in ZEN transformation. This conclusion is consistent with prior research [1]. Although GPx activity was markedly induced under ZEN exposure, GST remained unchanged. This may be due to the limited formation of electrophilic ZEN metabolites requiring conjugation, as glycosylation rather than glutathione conjugation serves as the dominant detoxification route in *H. zeae*.

#### 2.8.2. Determination of Intracellular Proline and Trehalose Content

Proline, a key compatible solute for maintaining cellular osmotic balance, functions as an osmoprotectant by stabilizing cellular structures and serves as a critical buffer against environmental stress. Under acidic conditions, it regulates ion imbalance mitigation and prevents cellular dehydration [53]. At pH 4, proline content in untreated groups increased significantly to 1.8 times (448.77 μg/mL) compared to the pH 7 controls, indicating successful acid stress adaptation in *H. zeae* (Figure 9G). However, ZEN-treated groups at pH 4 showed 4.3 times increase (296.23 μg/mL) over controls but a significant 33.99% reduction compared to untreated groups. Notably, at pH 7, ZEN exposure reduced proline by 72.37%. At 15 °C, ZEN-treated groups exhibited no significant proline change compared to the 28 °C controls, while untreated groups decreased to 47.39%of the control level. Proline accumulation under acidic stress was 3.5 times higher than that under hypothermic stress. Collectively, ZEN exposure significantly suppressed proline synthesis across all conditions, with maximal inhibition at physiological pH. This suppression likely stems from energy substrate competition: ZEN detoxification consumes ATP, while oxidative damage repair further depletes nicotinamide adenine dinucleotide phosphate (NADPH) [54], thereby compromising osmoprotective capacity.

Trehalose, a non-reducing disaccharide of two glucose units, exhibits high hydrophilicity and chemical stability. These properties establish it as a key stress protectant critical for maintaining cellular integrity under diverse stresses (e.g., acidity, hypothermia, oxidation, dehydration) and nutrient limitation [55]. Intracellular trehalose levels are correlated with yeast osmotolerance [17]. During hyperosmotic stress, extracellular hypertonicity causes water efflux and cellular dehydration, triggering rapid trehalose accumulation. Trehalose functions as an efficient osmolyte by balancing external osmotic pressure, maintaining turgor, and stabilizing macromolecular structures. At pH4, trehalose surged to 1787.2 μg/mL in ZEN-treated groups, likely attributable to combined membrane damage from acidity and ZEN-induced oxidative stress, triggering trehalose accumulation to counteract protein denaturation and lipid peroxidation (Figure 9H). This requires validation by TPS gene expression analysis. At pH 7, ZEN (5 μg/mL) induced no significant trehalose alteration, indicating that the stress it posed was insufficient to activate trehalose synthesis or disrupt homeostasis.

Upon ZEN exposure, the activities of SOD and CAT in *H. zeae* cells were significantly increased, while the contents of APX and proline also exhibited a moderate elevation. These changes indicate that ZEN induces an oxidative stress response in yeast cells. Notably, the activation of antioxidant defense mechanisms protects key metabolic enzymes from oxidative inactivation, thereby sustaining ZEN transformation through the glycosylation process. Similar adaptive responses have been reported in other toxin-detoxifying yeast [36,53], further confirming that oxidative stress regulation is an essential prerequisite for the continuous detoxification process.

## 3. Conclusions

This study identifies *Hannaella zeae*, a novel yeast from the Qinghai–Tibet Plateau, as a robust candidate for zearalenone (ZEN) detoxification, efficiently converting ZEN into less toxic glycosylated derivatives (ZEN-14-G and ZEN-16-G) while retaining significant activity under acidic and low-temperature stress. However, the current in vitro evidence and safety data from crude extracts necessitate further validation. Critical next steps include toxicological assessment of the purified metabolites, evaluation of degradation efficacy in complex feed and food systems, and a thorough safety analysis of the strain to support its development as a food-grade biocontrol agent.

## 4. Materials and Methods

### 4.1. ZEN Biotransformation Yeast Isolation and Identification

Zearalenone was purchased from Macklin Biochemical Co., Ltd. (Shanghai, China). The nutrient yeast dextrose agar (NYDA) consisted of 10 g/L of anhydrous glucose, 8 g/L of beef extract, 5 g/L of yeast powder, and 20 g/L of agar powder. The nutrient yeast dextrose broth (NYDB) was NYDA without agar. A portion (1–5 g) of soil samples or plant tissues was dissolved in 100 mL of sterile water. Its solution was oscillated at 200 rpm/min for 30 min. After being diluted, one hundred microliters of itwas spread on Bengal red medium plates from Guangdong Huankai Microbial Sci.&Tech. CO., Ltd. (Guangzhou, China), and were incubated at 28 °C for 3–5 d. Colonies exhibiting yeast morphological features were selected and streaked on NYDA plates. The purified yeast strains were inoculated into NYDB medium. The yeast cell concentration was adjusted to 1 × 10^8^ cells/mL and were transferred to a 50 mL conical flask containing sterile NYDB medium with 5 μg/mL ZEN [56]. They were cultured at 28 °C and 150 rpm/min for 48 h in a shaker, with sterile water as a control. Each treatment had three replicates. Then, the cultivated mixture was filtered through a 0.22 μm filter for high performance liquid chromatography (HPLC) analysis to evaluate the biodegradation effect.

The HPLC analysis was using a C18 column (4.6 mm × 250 mm; article size, 5 μm; Agilent Technology Inc, Santa Clara, CA, USA). The mobile A phase was 10% formic acid and the mobile phase B was pure methanol. The flow rate was set at 0.7 mL/min. Detection was accomplished using a fluorescence detector set at excitation and emission wavelengths of 274 nm and 440 nm, respectively [57,58].

### 4.2. Microstructure and Phylogenetic Identification of Candidate Yeast

Yeast cells for microstructure test were harvested during the logarithmic growth phase and fixed overnight at 4 °C in 2.5% glutaraldehyde solution after centrifugation to concentrate the samples. The fixed cells were then rinsed with phosphate-buffered saline (PBS)and dehydrated through a graded ethanol series (30%, 50%, 70%, 80%, 90%, and 100%). After dehydration, the cells were air-dried and observed using scanning electron microscopy (ZEISS, Oberkochen, Germany).

The phylogenetic identification was performed using universal primers to sequence the D1/D2 segment of the 26S rDNA gene derived from the chosen strains: forward primer NL1 (5′-GCATATCAATAAGCGGAGGAAAAG-3′) [59] and reverse primer NL4 (5′-GGTCCGTGTTTCAAGACGG-3′) [60]. The PCR products were verified by electrophoresis on a 1% agarose gel. The DNA fragments were recovered using the SanPrep column DNA gel recovery kit and sequenced by Sangon Bioengineering Co., Ltd. (Shanghai, China). The obtained 26S rDNA gene sequences were analyzed by using the BLASTN algorithm against the NCBI nucleotide collection (nr/nt) database. The phylogenetic tree was built using the neighbor-joining approach (bootstrap = 1000 replicates) by MEGA 11.0 software (Temple University, Philadelphia, PA, USA).

### 4.3. Biotransformation Effect of Tibetan Yeast on ZEN

#### 4.3.1. Effect of Tibetan Yeast Concentration on ZEN Biotransformation

After incubation, the yeast cells were suspended in sterile water and counted using a hemocytometer. The liquid concentration was adjusted to 1 × 10^6^, 1 × 10^7^, 1 × 10^8^, and 1 × 10^9^ cells/mL. Then, the ZEN standard liquid was added into medium with final concentration 5 μg/mL. The concentration of mycotoxins in the samples was measured using HPLC after 48 h incubation, where the peak areas of the mycotoxin before and after the reaction were used to calculate both the residual ZEN content and the corresponding transformation rate.

#### 4.3.2. Effect of ZEN Concentration on Biotransformation

The yeast cell suspension (1 × 10^8^ cells/mL) was added to 50 mL of NYDB medium, while sterile distilled water was used as a control. An appropriate amount of ZEN standard liquid was added and adjusted to the final concentration of 2, 5, 10, 15, 20 μg/mL. The concentration of mycotoxins in the samples was measured using HPLC after 36 h incubation.

### 4.4. Effect of Tibetan Yeast on Fusarium Graminearum Growth in Vitro

Firstly, ten microliters of yeast cell suspension (1 × 10^8^ cells/mL) was aseptically inoculated into Petri dishes, followed by 10 mL of molten PDA, and sterile distilled water was used as a control. All plates were solidified at room temperature and subsequently incubated at 28 °C for 24 h. Secondly, an additional 10 mL of PDA was uniformly overlaid onto the pre-solidified agar surface. Thirdly, when they became solid, a 5 mm diameter agar block of *Fusarium graminearum* excised from the actively growing margin was centrally positioned on each plate. They were cultured at 28 °C for 7 days, whose diameters were measured from the third day.

### 4.5. Effect of Different Yeast Treatments on ZEN Transformation

To investigate the transformation way of ZEN by the Tibetan yeast, ZEN residues of different yeast treatments were analyzed, which included yeast suspension at 1 × 10^8^ cells/mL concentration, heat killed yeast suspension (water bath at 100 °C for 30 min) cell-free filtrate and intracellular component treatment, while the sterile distilled water as a control. Then, ZEN was added at a final concentration of 5 μg/mL and the remaining ZEN content was quantified using HPLC.

### 4.6. Effects of Cycloheximide on ZEN Transformation

The effect of cycloheximide on the ZEN biotransformation by yeast was evaluated with modifications based on a previously described method [61]. Viable yeast cells were adjusted to a concentration of 1 × 10^8^ cells/mL and inoculated in 50 mL of NYDB medium with cycloheximide at the concentration of 2.5 μg/mL, while others were added sterile distilled water as a control. All samples were collected at 12 h intervals, and the residual ZEN concentration was determined by HPLC.

### 4.7. Transformation Product Analysis and Safety Evaluation

#### 4.7.1. Transformation Product Analysis

Chromatographic separation was conducted by the Ultimate 3000 UHPLC system (Thermo Fisher Scientific, Waltham, MA, USA). The injection volume, flow rate and column temperature were controlled at 2 μL, 0.3 mL/min and 40 °C, respectively. The mobile phase comprised water with 0.1% formic acid and 1 mM ammonium acetate (A) with MeOH (B). The detection was performed with an orbitrap mass spectrometer (Executive^TM^; Thermo Fisher Scientific, Waltham, MA, USA) equipped with an electrospray ion source (ESI). In the full MS/dd-MS2 mode, the mass scan range was 100–800 *m*/*z* with the resolving power set to 70,000 FWHM, AGC target 3e6, IT 100 ms, isolation window 1 *m*/*z*, and dynamic exclusion 5 s.

#### 4.7.2. Transformation Product Safety Evaluation

The *C. elegans* was used to evaluate the transformation products safety. The positive control was the filtrate of the culture medium containing 5 μg/mL ZEN without yeast, while the treatment was the filtrate of the culture medium co-cultured yeast with 5 μg/mL ZEN for 36 h. The filtrate of the culture medium added sterile distilled water was the control.

The fecundity of *C. elegans* was evaluated based on the quantity of offspring. The synchronized (L1) *C. elegans* larvae were transferred to a new nematode growth medium (NGM) containing sufficient nutrients and different concentrations of ZEN, which were incubated at 20 °C to the adult stage. Then, adult *C. elegans* were taken into 96-well plates containing M9 buffered solution. The quantity of eggs at each stage was counted under a XTZ-binocular microscope (Shanghai Puda Optical, Hangzhou, China) after 72 h incubation. After synchronization, the L1-stage *C. elegans* were added to NGM with inactivated *Escherichia coli* OP50, which was chronically exposed under medium with ZEN and ones biodegraded by Tibetan yeast when the *C. elegans* were at the adult stag Their body length was recorded by an orthostatic fluorescence microscope (Nikon Corporation, Tokyo, Japan) from larval (L1–L4) to adult phase and measured using Image (win64 version) analyze software. Each treatment had three replicates and each replicate contained 10 nematodes.

### 4.8. Tolerance of Tibetan Yeast to Acid and Temperature Stress

For acid stress tolerant test, the Tibetan yeast and *S. cerevisiae* (control) suspension with a concentration of 1 × 10^8^ cells/mL was added to 50 mL NYDB medium with a pH value of four (pH 4) and cultured at for 60 h at 200 rpm/min and 28 °C. Yeasts were taken out every 12 h and spread on NYDA medium after dilution. For temperature stress tolerant test, Tibetan yeast was incubated at 4 °C, 28 °C, and 45 °C for 3 h in NYDB medium with a concentration of 1 × 10^8^ cells/mL. After being treated, the yeast culture medium was taken out and spread on NYDA medium, whose colonies were counted after 2 days incubation at 28 °C. Each treatment had three replicates.

### 4.9. Related Substances Variation in Yeast to ZEN and Stress

#### 4.9.1. Determination of the Correlation Enzyme Activity

To evaluate the oxidative stress response of yeast cells under different environmental conditions, including varying temperatures, pH levels, and ZEN exposure, MDA content and the activities of key antioxidant enzymes were measured. MDA, a classical marker of lipid peroxidation, was quantified to assess the extent of membrane lipid oxidation and cellular damage. Elevated MDA levels indicated enhanced lipid peroxidation and membrane damage, particularly under ZEN stress. In contrast, the upregulation of SOD, CAT, ascorbate peroxidase (APX), glutathione peroxidase (GPX), and glutathione s-transferase (GST) activities reflected the activation of the cellular antioxidant defense system. Specifically, SOD catalyzes the conversion of superoxide radicals into H_2_O_2_, while CAT, APX, and GPX decompose H_2_O_2_ to less toxic products, thereby alleviating oxidative damage. Meanwhile, GST contributes to detoxification through glutathione conjugation with xenobiotic compounds. Yeast cell lysates were prepared using extraction buffers from activity assay kits by Grace Biotechnology Co., Ltd. (Suzhou, China). After centrifugation at 12,000× *g* for 10 min at 4 °C, supernatants were assayed for CAT, SOD, APX, MDA, GST, and GPx activities in 96-well plates at different absorbance values using a microplate reader (Thermo, USA).

#### 4.9.2. Determination of Intracellular Proline and Trehalose Content

To evaluate the osmoprotective response of *Hannaella zeae* under various stress conditions, intracellular trehalose and proline levels were measured. Yeast cultures were exposed to different temperatures, pH values, and the presence or absence of ZEN. Trehalose content was quantified based on its enzymatic conversion to glucose using a commercial trehalose assay kit by Grace Biotechnology Co., Ltd. (Suzhou, China). In contrast, proline accumulation was determined following the anthrone colorimetric method by Grace Biotechnology Co., Ltd. (Suzhou, China). Absorbance readings were recorded using a microplate reader at the specified wavelengths. These measurements enabled the assessment of yeast metabolic adjustments and osmoprotective responses under diverse environmental and toxin-induced stresses. For proline analysis, supernatants were incubated in a 90 °C shaking water bath for 10 min, cooled to room temperature, centrifuged at 8000× *g* for 10 min at 25 °C. After shaking at room temperature for 30 min, the sample was centrifuged under the same conditions. The supernatant was then discarded prior to the measurement of trehalose content. Trehalose content was determined using an anthrone-sulfuric acid method adapted for a 96-well microplate format. Briefly, each well received the sample, distilled water, and Reagent 1 in sequence. The plate was then incubated in a boiling water bath (95–100 °C) for 3 min. After cooling to room temperature, the solution was mixed thoroughly, and the absorbance was measured at 620 nm using a microplate reader (Thermo, USA). Each well was sequentially loaded with the sample, distilled water, Reagent 1 and glacial acetic acid. The plate was then incubated in a water bath at 95 °C for 30 min. After cooling to room temperature, absorbance was immediately measured at 520 nm using a microplate reader (Thermo, USA). The proline concentration was calculated according to the formula provided in the manufacturer’s instructions.

### 4.10. Data Treatment and Statistical Analysis

All datasets were formally assessed for normality using the Shapiro–Wilk test. After confirming that the data met the assumptions for parametric analysis, one-way analysis of variance (ANOVA) was applied. For datasets where ANOVA indicated significant differences, Tukey’s honest significant difference (HSD) post hoc test was performed for multiple comparisons. All statistical analyses were conducted using SPSS software (version 26.0, IBM, Armonk, NY, USA). Figures were drawn by the Prism 8.0 software (GraphPad Software, Inc., San Diego, CA, USA). Data were presented as mean ± SD. Results with a *p*-value < 0.05 were considered statistically significant.

## Data Availability

The original contributions presented in this study are included in the article. Further inquiries can be directed to the corresponding author(s).

## Abbreviations

The following abbreviations are used in this manuscript:

APX	ascorbate peroxidase
*C. elegans*	*Caenorhabditis elegans*
CAT	catalase
GPx	glutathione peroxidase
GST	glutathione s-transferase
H_2_O_2_	hydrogen peroxide
HPLC	high performance liquid chromatography
MDA	malondialdehyde
NADPH	nicotinamide adenine dinucleotide phosphate
NGM	nematode growth medium
NMR	nuclear magnetic resonance
NYDA	nutrient yeast dextrose agar
NYDB	nutrient yeast dextrose broth
PCR	polymerase chain reaction
PBS	phosphate-buffered saline
PDA	potato dextrose agar
ROS	reactive oxygen species
*S. cerevisiae*	*Saccharomyces cerevisiae*
SEM	scanning electron microscopy
SOD	superoxide dismutase
TPS	trehalose-6-phosphate synthase
UHPLC-Q-Orbitrap-HRMS	ultra-high performance liquid chromatography quadrupole-orbitrap high-resolution mass spectrometry
ZEN	zearalenone
ZEN-14-G	zearalenone-14-β-D-glucopyranoside
ZEN-16-G	zearalenone-16-β-D-glucopyranoside
